# Environmental, Human and Ecotoxicological Impacts of Different Rice Cultivation Systems in Northern Thailand

**DOI:** 10.3390/ijerph20032738

**Published:** 2023-02-03

**Authors:** Patharanun Toolkiattiwong, Noppol Arunrat, Sukanya Sereenonchai

**Affiliations:** Faculty of Environment and Resource Studies, Mahidol University, Nakhon Pathom 73170, Thailand

**Keywords:** rice cultivation, carbon footprint, water footprint, human health, ecotoxicological

## Abstract

Sustainable practices in rice cultivation require effective farming management concerning environmental and human health impacts. In this study, three rice cultivation systems, namely low-land, upland, and terraced rice in the Mae Chaem District, Chiang Mai Province, were assessed and the carbon footprint (CF), water footprint (WF), and human and ecotoxicological impacts were compared from pesticide application. The results showed that the highest CF intensity was observed in terraced rice with 1.15 kg CO_2_eq kg^−1^ rice yield, followed by lowland rice (1.02 kg CO_2_eq kg^−1^ rice yield) and upland rice (0.17 kg CO_2_eq kg^−1^ rice yield) fields. Moreover, lowland rice cultivation generated the highest total WF with 1701.6 m^3^ ton^−1^, followed by terraced rice (1422.1 m^3^ ton^−1^) and upland rice (1283.2 m^3^ ton^−1^). The lowland rice fields had the most impact on human health and freshwater ecotoxicity, followed by the terraced and upland rice cultivation systems. The results also showed that most of the pesticides remaining in soils were chlorpyrifos (98.88%), butachlor (96.94%), and fipronil (95.33%), respectively. The substances with the greatest distributions in freshwater were acephate (56.74%), glyphosate (50.90%), and metaldehyde (45.65%), respectively. This study indicated that, with more agricultural inputs, higher CF, WF, human health impacts, and freshwater ecotoxicity were generated. Although the use of pesticides in the study areas did not exceed the recommendations on the packaging, glyphosate and chlorpyrifos are restricted in Thailand, so it is necessary to monitor their use due to their long-term health effects.

## 1. Introduction

Based on the geographical distribution in Thailand, traditional rice cultivation practices can be simply classified into lowland, upland, and terraced rice. Lowland rice is usually grown in floodplain areas where fields are flooded during the growing season. Cultivation can be done more than once a year [1]. Upland rice is generally grown on hills and sloping areas where water relies only on rainfall, leading to cultivation once a year. Terraced rice is also grown on high hills and sloping areas where natural water is available, facilitating fields that are flooded during the growing season [2]. Among these rice cultivation systems, management practices are different throughout the rice’s life cycle, which causes the difference in environmental and human health impacts [3,4]. 

Carbon footprint (CF) and water footprint (WF) were developed to assess the pressure from human activities on the environment. For climate change, the CF assessment has been widely used to express a quantity of carbon dioxide equivalent (CO_2_eq) [5], which is associated with the sum of three main greenhouse gases’ (GHGs: CH_4_, N_2_O, and CO_2_) contributions to global warming. In rice paddies, methane (CH_4_) is produced in continuous flooding, which generates an anaerobic environment and favors methanogen bacteria for CH_4_ generation [6]. Nitrous oxide (N_2_O) can be directly and indirectly produced from chemical fertilizers, manure management, organic fertilizers, and crop residue burning, which is mostly emitted during dry periods [7]. Excessive usage of nitrogen fertilizer significantly increases denitrification from paddy soils [8]. CO_2_ is mainly generated from fuel combustion (diesel and gasoline fuels) during the cultivation stages [9]. To mitigate CO_2_ emission, paddy soils have the potential for soil carbon sequestration through management practices (e.g., incorporating rice straw and stubble, manure and compost application, green manure, organic fertilizer, and minimum/no tillage) [10,11,12,13]. Thus, it is necessary to take soil organic carbon (SOC) into account when estimating CF [10,14,15]. Moreover, WF is used as an indicator for measuring the volume of direct and indirect water use in the life cycle of production, which consists of green, blue, and gray WFs [16]. Green WF indicates the amount of rainwater stored in the soil that is evaporated, transpired, and consumed by plants during cultivation. Blue WF refers to the water consumption from rivers, lakes, ponds, and groundwater, while gray WF means the consumption of freshwater to dilute a load of pollutants and meet the standard of water quality [17,18].

Pesticides cause serious concerns for human health impacts. Once pesticides are applied, pesticide residues remain in the soil and can be transported and bioaccumulated in the air, crops, and food webs [19,20]. Safi [21] reported that lung cancer; breast cancer; lymphomas; leukemia; and cancers of the urinary bladder, prostate, brain, colon, stomach, liver, and thyroid gland are closely correlated with chronic exposure to pesticides in the Gaza Governorates. Ghafouri-Khosrowshahi et al. [22] found that rural farmers who live close to agricultural areas have higher health risks than urban people. They also concluded that exposure to organophosphate pesticides may impair hypothalamic and pituitary endocrine functions and gonadal processes. To evaluate the total human population damage, disability-adjusted life years (DALYs) are usually used to express the years of life lost [23]. Meanwhile, a potentially disappeared fraction of species (PDF) is a relative measure of the damage to ecosystem quality, indicating a fraction of species richness that can be potentially lost due to an environmental mechanism [24]. Thus, it is very important for minimizing the pesticides used in agricultural lands to protect human health, which needs a comprehensive environmental and human health impact assessment.

Chiang Mai Province is a mountainous area in Northern Thailand with steep river valleys, upland areas, and lowland rural areas. Such a geographical pattern causes different traditional ways of life for rice cultivation, consisting of upland, terrace, and lowland rice. To date, there is no comprehensive study to assess and compare the environmental and human health impacts of these rice cultivation systems. Therefore, the objectives of this study were to (1) evaluate the CF and WF and (2) assess the human health impact of pesticide application. This study provides scientific insight for policy planning and advocacy to reduce environmental damage and human health issues.

## 2. Materials and Methods

### 2.1. Study Area

The study was conducted in Mae Chaem District, Chiang Mai Province, Northern Thailand, with a wide range of elevation from 282 to 2565 m.a.s.l. at the highest peak (Doi Inthanon) [25]. The climate is classified as a tropical climate with three seasons (rainy May–October, winter November–February (10–19 °C), and summer January–April) [26]. The average annual rainfall was 978.2 mm, with the highest rainfall being in August, and an average annual temperature of 25.4 °C, with the hottest month being April (40 °C) [27]. A slope complex series is classified for the soils in the highlands (slope > 35%) of Thailand [28]. The soil is mainly mostly reddish-brown lateritic. The soil textures are mostly sandy clay loam, sandy clay, and clay loam, with an acidic soil pH (5.0–5.8) [29].

**Figure 1:**
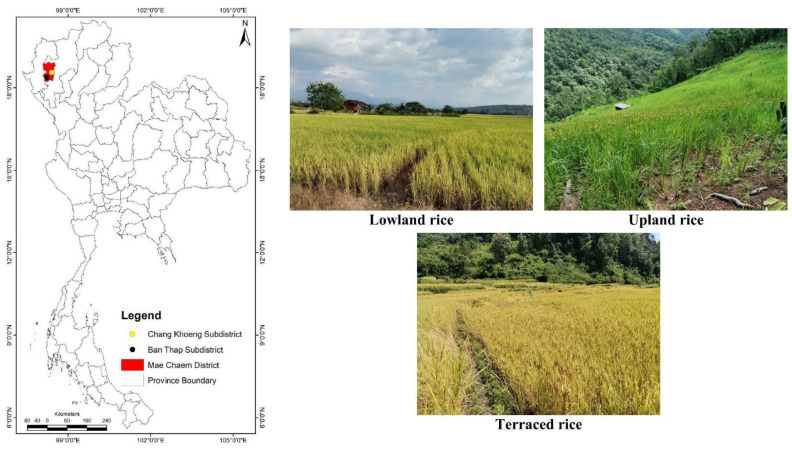
Study area.

In this study, lowland rice cultivation was investigated in the Chang Khoeng Subdistrict, while upland and terraced rice fields were studied in the Ban Thap Subdistrict, Mae Chaem District, Chiang Mai Province (Figure 1).

### 2.2. Data Collection

#### 2.2.1. Farm Management Practices 

Three crop years (2019/2020–2021/2022) of farm practices were obtained from the owners of upland (47 farms), terraced (30 farms), and lowland (66 farms) rice farms using a questionnaire. All the practices were collected throughout the crop year, including seeds, fossil fuels (diesel and gasoline), manure, chemical fertilizers (N-P_2_O_5_-K_2_O), insecticides, herbicides, labor, and rice yields (Table 1). In addition, dates and months of all practices were recorded, consisting of land preparation, sowing, transplanting, chemical fertilizer application, insecticide and herbicide application, water pumping, and harvesting.

#### 2.2.2. Soil Sampling and Analysis

Soil samples from three rice systems were collected at 0–30 cm in depth after the harvests for three consecutive years (2020–2022). At each field, soil samples were randomly gathered from five pits. At each pit, soil samples were collected for three replications. Undisturbed soil cores (5.0 cm width × 5.5 cm length) were used to collect soil samples; then, the soil bulk density was measured after drying in an oven at 105 °C for 24 h.

After air drying soil samples at room temperature for 7–10 days, soil samples were crushed and passed through a 2 mm sieve. Organic carbon (OC) was determined by potassium dichromate (K_2_Cr_2_O_7_) oxidation [30].

#### 2.2.3. Soil Organic Carbon Calculation

The SOC stock was calculated using the following equation:

(1) $$SOC_{30cm}=\left(\rho \times OC\times L\right)\times 1000$$

(2) $$\Delta SOCS_{30cm}=\frac{SOC_{30cm\left(2022\right)}-SOC_{30cm\left(2020\right)}}{2}\times \frac{44}{12}$$

where *SOC*_30_*_cm_* is soil organic carbon stock (kg C ha^−1^), *ρ* is soil bulk density (g cm^−3^), *OC* is organic carbon content (%), L is soil thickness (cm), $\Delta SOCS_{30cm}$ is the SOC sequestration at a depth of 30 cm (kg CO_2_eq ha^−1^ year^−1^), $SOC_{30cm\left(2022\right)}$ and $SOC_{30cm\left(2020\right)}$ are the amounts of SOC stocks at a depth of 30 cm (kg C ha^−1^) in 2022 and 2020, respectively, and 44/12 is the coefficient for converting C into CO_2_.

Due to soil bulk density changes over time, the soil thickness (cm) was adjusted by using the equivalent soil mass method to reduce the error in the SOC stock calculations. The equation is used below [31]:

*Soil mass=ρ* × *L* (3)

where soil mass is the mass of the soil sample (kg soil m^−2^). In this study, the soil mass values in 2020 were from the beginning of the study, while the soil mass values in 2022 were from the end of the study.

### 2.3. System Boundary and Functional Unit

The CF was calculated following the 2019 Refinement to the 2006 IPCC Guidelines for National Greenhouse Gas Inventories [32]. The cradle-to-gate concept was used for the life cycle assessment of rice production, including the raw material production, transportation, field emissions, and harvesting stages. Emissions of CO_2_, CH_4_, and N_2_O were expressed in carbon dioxide equivalents (CO_2_eq). The radiative forcing potential relative to CO_2_ was 28 for CH_4_ and 265 for N2O [33]. The functional unit of CF is expressed as kg CO_2_eq ha^−1^ year^−1^ and kg CO_2_eq kg^−1^ rice yields.

### 2.4. Carbon Footprint Calculation

In this study, the equations from the 2019 Refinement to the 2006 IPCC Guidelines for National Greenhouse Gas Inventories [32] were used as follows:

(4) $$CFI=\frac{GHG_{total}-\Delta SOCS_{30cm}}{Y}$$

(5) $$GHG_{total}=GHG_{rm}+GHG_{tran}+GHG_{uti}+GHG_{CH_{4}}+GHG_{N_{2}O}$$

where *CFI* is the carbon footprint intensity (kg CO_2_eq kg^−1^ rice yield), *GHG_total_* is the total GHG emissions (kg CO_2_eq ha^−1^ year^−1^), *Y* is the rice yield (kg ha^−1^ year^−1^), $GHG_{rm}$ is GHG emissions from the raw material production (kg CO_2_eq ha^−1^ year^−1^), $GHG_{tran}$ is GHG emissions from transportation activity (kg CO_2_eq year^−1^), $GHG_{uti}$ is GHG emissions during the utilization phase (kg CO_2_eq ha^−1^ year^−1^), $\mathrm{and}\;GHG_{CH_{4}}$ and $GHG_{N_{2}O}$ are the methane (kg CH_4_ ha^−1^) and the direct and indirect N_2_O emissions (kg N_2_O ha^−1^) from rice cultivation. All the emission factors were obtained from sources by Arunrat et al. [1] and Arunrat et al. [15]. The emissions factor of human labor was 0.86 kg CO_2_eq day^−1^ [34].

### 2.5. Water Footprint Calculation

The total WF in rice cultivation (*WF_total_*, m^3^ ton^−1^) is the sum of green, blue, and gray water [35,36,37], as presented in Equation (6).

(6) $$WF_{total}=WF_{green}+WF_{blue}+WF_{gray}$$

The green WF and blue WF are calculated using Equations (7)–(10), respectively.

(7) $$WF_{green}=\frac{CWU_{green}}{Y}=\frac{10\times \sum_{d=1}^{lgp}ET_{green}}{Y}$$

(8) $$WF_{blue}=\frac{CWU_{blue}}{Y}=\frac{10\times \sum_{d=1}^{lgp}ET_{blue}}{Y}$$

(9) $$ET_{green}=min\left(ET_{c},P_{eff}\right)$$

(10) $$ET_{blue}=max\left(0,ET_{c}-P_{eff}\right)$$

where *CWU* is crop water use (m^3^ ha^−1^), *ET_green_* is evapotranspiration of green water (mm day^−1^), *ET_blue_* is evapotranspiration of blue water (mm day^−1^), *lgp* is the growing period, *P_eff_* is the effective rainfall, and *ET_c_* is the crop evapotranspiration. The “0” value is considered when *P_eff_* exceeds crop evapotranspiration. Evapotranspiration was calculated by the CROPWAT 8.0 model.

The gray WF calculation (*WF_gray_,* m^3^ ha^−1^) used Equation (11) [38]:

(11) $$WF_{gray}=\frac{\alpha \times \left(\sum_{x=1}^{n}N+ON\right)}{\left(C_{max}-C_{nal}\right)/Y}$$

where α is the fraction of leaching runoff (0.1 is for nitrogen) [39], *ON* is the amount of organic amendment (kg N ha^−1^), *C_max_* is the maximum acceptable concentration of a load of pollutant (NO_3_-N < 5 mg L^−1^, Pollution Control Department [40]), and *C_nal_* is the natural N concentration (*C_nal_* = 0 kg m^−3^).

### 2.6. Human and Ecotoxicity Impacts

This study followed a method of life cycle impact assessment to assess the health and freshwater ecotoxicity impacts of pesticide use in three rice planting systems (lowland rice, upland rice, and terraced rice fields) using the USEtox model. This study examined the proportion of pesticides as an emission in agricultural soils at steady-state conditions.

The USEtox model was developed under UNEP–SETAC as a model for determining the health effects and environmental toxicity of chemicals [41]. Fate transportation in days, exposure, and effects from intakes is a process for determining the effects in the USEtox model. The human health and ecotoxicity impact scores were determined by multiplying the released pesticide mass, depending on active ingredients coupled with the characterization factor provided by USEtox. Accordingly, the human toxicity and freshwater ecotoxicity impact scores (IS) can be described as:

(12) $$IS=\underset{i,x}{\sum }M_{x,i}\times CF_{x,i}$$

where $M_{x,i}$ is the mass of pesticide active ingredient *x* emitted to compartment *I* per area treated (kg _emitted_ ha^−1^), and $CF_{x,i}$ is the amount of pesticide active ingredient *x* released to compartment *i* expressed as disability-adjusted life years (DALYs). For the freshwater ecotoxicity impact score, a potentially disappeared fraction of species (PDF) was used to demonstrate the impact by consideration between the freshwater volume (m^3^) and the duration of one day per kg pesticide active ingredient emission (PDF m^3^ day kg^−1^_emitted_). The characterization factor for human health and freshwater ecotoxicity impacts can be calculated as Equations (13) and (14).

(13) $$CF_{x}=IF_{x}\times EF_{x}\times DF_{x}$$

(14) $$IF_{x}=FF_{x}\times XF_{x}$$

The characterization factor for human health represents health effects at the endpoint level (DALY kg^−1^_emitted_). The characterization factor for each pesticide is related to the human intake fraction ($IF_{x}$) via the inhalation and ingestion route (kg_intake_ kg^−1^_emitted_), where fate ($FF_{x}$) and human exposure ($XF_{x}$) matrices can be integrated into an intermediary matrix referred to as the intake fraction. $EF_{x}$ is the health effect of pesticide intake (cases kg^−1^_intake_), and $DF_{x}$ is the damage occurrence (DALY case^−1^).

The characterization factor for freshwater ecotoxicity is due to a combination of fate transportation ($FF_{x}$) (day). The $XF_{x}$ is the exposure of the bioavailable fraction of pesticides in freshwater, $EF_{x}$ represent ecological effects of changes in a potentially affected fraction (PAF m^3^ kg^−1^), and $DF_{x}\;$is the damage factor (PDF PAF^−1^). The characterization factors for nearly 4000 chemicals were included in the USEtox model.

### 2.7. Statistical Analysis

The analyses were performed using SPSS (v. 25.0). A one-way ANOVA and Tukey’s honestly significant difference (HSD) tests (*p* < 0.05) were performed to identify differences in values among the three rice cultivation systems.

## 3. Results

### 3.1. Input Inventory Analysis

As shown in Table 1, it is noteworthy that the largest input of seed can be seen in the lowland rice system with 137.5 (±50) kg ha^−1^ crop^−1^. There was no significant difference in seed input among the three rice systems. However, the amount of gasoline used in the rice systems is significantly different (*p* < 0.05). Similar to seeds, the gasoline input took the largest portion of the lowland rice system, with 125.0 (±43.75) L ha^−1^ per crop, whereas nearly half of the amount used in the lowlands was utilized in terraced rice. During the study period, there was no remarkable difference in the use of diesel between lowland and terraced rice, while no diesel input was found in the upland rice system. It is interesting to note that the agricultural input of straw was significantly different among the three rice systems (*p* < 0.05). The lowland rice system used straw 3474.4 (±1250) kg ha^−1^ per crop, terraced rice 2618.5 (±1250) kg ha^−1^ crop^−1^, and upland rice 1316.7 (±625) kg ha^−1^ per crop, respectively. In lowland and terraced rice systems, no significant difference in manure input was noteworthy, whereas farmers did not use manure in the upland rice system. On the other hand, terraced rice was labor-intensive, with the need for 5.0 (±3.0) persons per day, while rice farming in the upland area had a lesser labor need (3.0 ± 2.0 persons per day). The input of 16-20-0 fertilizer in lowland and terraced rice was slightly different, and the upland rice does not use such a kind of fertilizer. However, 16-16-8 fertilizer was utilized in all rice systems. The same amount of that kind of fertilizer was used in lowland and terraced rice farming, although upland rice needed a smaller amount of 16-16-8 fertilizer compared to the other two rice systems. The use of 46-0-0 fertilizer was not popular in the study area. Only lowland rice used that fertilizer, and the estimated amount was 62.5 (±37.25) kg ha^−1^ crop^−1^.

### 3.2. Soil Organic Carbon Stock and Sequestration

The soil organic carbon (SOC) stock was significantly higher in the upland rice system compared to the other two rice systems in the three selected years (2020, 2021, and 2022) (*p* < 0.05). On the other hand, the SOC stock in each rice system was not significantly different during the study period. In 2022, the upland rice stored 61,068.6 ± 867 kg C ha^−1^ of SOC, terraced rice 44,960.5 ± 1045 kg C ha^−1^, and lowland rice 32,804.7 ± 1127 kg C ha^−1^, respectively. It is noteworthy that, in each year of the study period, the SOC stocks in the three rice systems were significantly different (*p* < 0.05). The present study also proved that the SOC storage capacity of the lowland rice system was nearly half that of the upland system. Moreover, the differences in SOC sequestration (ΔSOCS) during the study period were more significant in the lowland rice system compared to the other two systems, with an increase of 52.9 (±12.2) kg C ha^−1^ year^−1^ and 194.0 (±45.1) kg CO_2_eq ha^−1^ year^−1^ (Table 2).

### 3.3. Greenhouse Gas Emissions and Carbon Footprint Intensity

Among the agricultural inputs at the raw materials production stage, chemical fertilizers contributed to the highest GHG emission compared to the other inputs, with 686.5 ± 181.5 kg CO_2_eq ha^−1^ year^−1^ in lowland rice, 315.6 ± 93.6 kg CO_2_eq ha^−1^ year^−1^ in terraced rice, and 125.7 ± 24.6 kg CO_2_eq ha^−1^ year^−1^ in upland rice, respectively. It is also noteworthy that the emissions due to chemical fertilizers in different rice systems were significantly different (*p* < 0.05), and lowland rice made the greatest amount of emissions. At the same stage, herbicides and pesticides took second and third place in contributing to GHG emissions. Farming lowland rice caused the highest level of emission with 82.4 (±10) kg CO_2_eq ha^−1^ year^−1^ due to herbicides, while the use of pesticides in terraced rice made the emission of 97.5 ± 10.1 kg CO_2_eq ha^−1^ year^−1^. Upland rice did not use gasoline and diesel, so it made zero emissions due to these inputs. However, these two agricultural inputs significantly contributed to GHG emissions at the field emission stage in lowland and terraced rice (*p* < 0.05). On the other hand, the contribution of CH_4_ in lowland and terraced rice farming at the cultivation stage made the highest level of emissions, with 2767.9 ± 1512.4 kg CO_2_eq ha^−1^ year^−1^ and 2914.3 ± 1604.5 kg CO_2_eq ha^−1^ year^−1^, respectively. Herbicides, N_2_O, and labor were also sources of emissions, and they significantly differently contributed to GHG emissions in the three different rice systems (*p* < 0.05). In harvesting rice, zero emissions were found in terraced and upland rice systems. In total and net GHG emissions, lowland rice farming took first place. Similarly, the lowland rice system gave the highest yield with 4632.5 ± 870.6 kg ha^−1^ year^−1^. The highest carbon footprint intensity was observed in terraced rice with 1.15 (±0.66) kg CO_2_eq kg^−1^ rice yield, and the intensities in the three rice systems were significantly different (*p* < 0.05) (Table 3).

### 3.4. Water Footprint

In the three rice systems, the consumption of green WF was obviously high compared to blue and gray WFs. Upland rice took first place in using green WF with 94.9% of the total WF, while the only remaining WF in the same rice system was gray WF. However, the amount of green WF in the three different rice systems was not significantly different, whereas a remarkable difference in gray WF of each system was found. It is interesting to note that lowland rice generated the highest amount of gray WF with 589.7 (±125.7) m^3^ ton^−1^ among the three systems while it used green water with 59.3% of total WF. The consumption of blue WF was comparatively low in the terraced and lowland rice systems compared to the other WFs, and no blue WF was found in upland rice (Table 4).

### 3.5. Pesticide Usage in Three Rice Planting Systems

This study found that farmers used a total of 14 pesticides, including six herbicides: 2,4-D dimethyl ammonium, acetochlor, alachlor, atrazine, butachlor, and glyphosate; five insecticides: acephate, carbendazim, chlorpyrifos, cypermethrin, and fipronil; two fungicides: captan and mancozeb; and one molluscicide: metaldehyde. The areas in the order of the greatest use of pesticide types and amounts were lowland rice fields, upland rice, and terraced rice systems, respectively. The amounts of active ingredients used per hectare per year differed in each area, as shown in Table 5.

This study compared farmers’ pesticide uses to the maximum recommended dosages on each package per hectare per year. Alachlor, atrazine, and 2,4-D dimethyl ammonium were the top three pesticides used in 2020, 2021, and 2022. It was found that most of the farmers used pesticides as recommended, except for acephate and glyphosate, for all three years in the lowlands; carbendazim in 2020 in the uplands; and carbendazim in 2020, captan in 2021, and fipronil in 2021 at cultivation, used in doses higher than the instructions on the package. In addition, the use of pesticides in rice cultivation for acetochlor, atrazine, butachlor, and metaldehyde tends to increase every year. Although the use of pesticides by farmers did not exceed the recommended dosage on the packaging, some substances must be monitored. Glyphosate is restricted in Thailand, making it necessary to monitor its use due to its long-term health effects. Likewise, chlorpyrifos is a pesticide listed as a type 4 hazardous substance in Thailand, and it was officially banned in 2020. This may require a review of the use of such pesticides in rice cultivation. Farmers in upland and terraced rice cultivation use fewer types of pesticides than lowland rice cultivation; in the lowland, the main purpose is commercial and focuses on good yields, while upland and terraced rice cultivation mostly grow rice for household consumption and sometimes for sale. In each area, there are agricultural scholars to give advice on the type and amount of pesticide use. However, it is not possible to control the type and amount of use as it should in reality. The accessibility of pesticides varies from area to area, resulting in different pesticides being used, such as atrazine, acetochlor butachlor, and glyphosate being used only in lowland cultivation. In addition, farmers in some areas have concerns about health issues, especially the use of glyphosate, which has a relatively high health impact due to its use restrictions in Thailand, and noticed that it was only used in the lowland cultivation area. All of this is the reason why the types of pesticides farmers use vary from area to area.

### 3.6. Toxicity from Pesticide Usage

The most noticeable impact on the amount of pesticide use was in lowland rice fields. The average impact score of pesticide use in rice cultivation showed that acephate had the greatest human health impact (non-cancer) at 9.39 × 10^−6^ DALYs, followed by atrazine at 1.76 × 10^−6^ DALYs and 2,4-D at 7.36 × 10^−6^ DALYs, respectively. On the other hand, atrazine showed a higher health impact (cancer) (6.46 × 10^−6^ DALYs) than acephate (1.44 × 10^−8^ DALYs). However, if pesticides do not show cancer and non-cancer health effects, it does not mean that these pesticides do not have that effect, as toxicological data may not be able to indicate such effects due to the lack of toxicological data on individual substances. Specifically, for their health effects, only two pesticides, atrazine and acephate, have toxicological data in the USEtox database. In upland rice and terraced rice systems, pesticides are not used as much, and therefore, the effects may not be apparent. In addition, the use of pesticides by most farmers did not exceed the recommendations on the packaging, resulting in low health effects.

**Figure 2:**
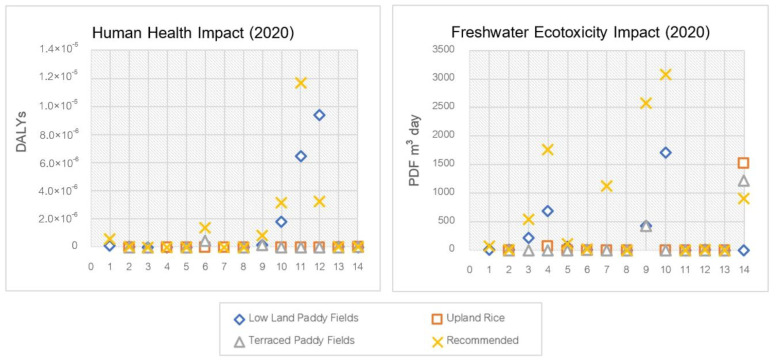
Human health and ecotoxicity impact scores on pesticide use in rice cultivation in 2020. 1. Chlorpyrifos, 2. Glyphosate, 3. Acephate, 4. Alachlor, 5. Butachlor, 6. 2,4-D dimethyl ammonium, 7. Fipronil, 8. Metaldehyde, 9. Mancozeb, 10. Atrazine, 11. Atrazine (cancer), 12. Acephate, 13. Acephate (cancer), and 14. Carbendazim.

In lowland rice fields, atrazine also showed the most freshwater ecotoxicity impact at 1702.79 PDF.m^3^.day due to specific toxicity data and the amount of substance used by farmers, followed by alachlor at 686.17 PDF.m^3^.day and mancozeb at 429.16 PDF.m^3^.day, respectively. In upland rice areas, carbendazim had the highest freshwater ecotoxicity impact at 1522.18 PDF.m^3^.day; as with the terraced rice farming, carbendazim also shows the highest toxicity at 1217.75 PDF.m^3^.day, followed by mancozeb at 429.16 PDF.m^3^.day, respectively. The human health and freshwater ecotoxicity impacts of rice cultivation (2020) are shown in Figure 2. However, the health impacts between cancer and non-cancer were calculated separately. The non-cancer health impacts were considered with NOEL (no observable effect level) or LOEL (lowest-observed effect level) to ED50 (effective dose at 50%), which differed from the cancer effect that was determined by a separate dose–response curve, depending on each of the toxicology test results for each substance.

**Figure 3:**
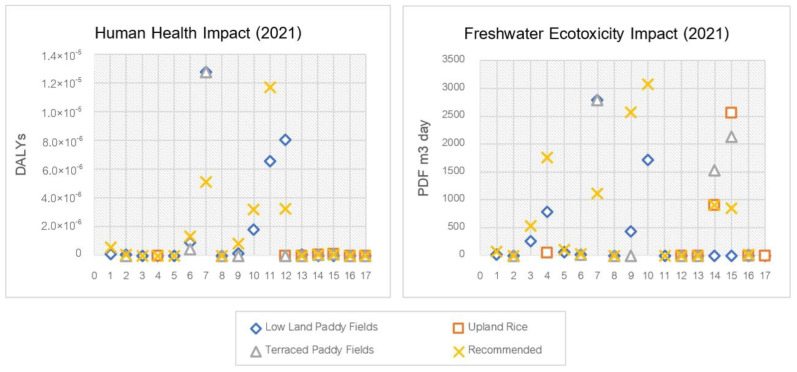
Human health and ecotoxicity impact scores on pesticide use in rice cultivation in 2021. 1. Chlorpyrifos, 2. Glyphosate, 3. Acephate, 4. Alachlor, 5. Butachlor, 6. 2,4-D dimethyl ammonium, 7. Fipronil, 8. Metaldehyde, 9. Mancozeb, 10. Atrazine, 11. Atrazine (cancer), 12. Acephate, 13. Acephate (cancer), 14. Carbendazim, 15. Cypermethrin, 16. Captan, and 17. Captan (cancer).

In 2021, the amount of pesticide used in each area changed, resulting in increased health impacts from the use of certain substances shown in Figure 3. Fipronil showed the most pronounced health impact (non-cancer) at 1.28 × 10^−5^ DALYs in both the lowland rice fields and terraced rice, followed by acephate (8.05 × 10^−6^ DALYs) and atrazine (1.77 × 10^−6^ DALYs), respectively. For cancer effects, atrazine (6.51 × 10^−6^ DALYs) also showed a greater effect than acephate (1.23 × 10^−8^ DALYs).

The freshwater ecotoxicity impact was more apparent in 2020, when initiating the use of certain pesticides, especially fipronil and cypermethrin, increased their impacts. In lowland rice fields, fipronil showed the greatest effect at 2801.46 PDF.m^3^.day, followed by atrazine at 1717.44 PDF.m^3^.day and alachlor at 777.19 PDF.m^3^.day, respectively. The freshwater ecotoxicity impact in the upland rice area stands out from the use of cypermethrin (2564.41 PDF.m^3^.day) and carbendazim (913.31 PDF.m^3^.day). In terraced rice farming, fipronil showed the highest freshwater ecotoxicity impact at 2801.46 PDF.m^3^.day, followed by cypermethrin at 2137.00 PDF.m^3^.day and carbendazim at 1522.18 PDF.m^3^.day, respectively.

In 2022, the health impacts were still quite pronounced in lowland rice fields. Acephate showed the greatest health impact (non-cancer) at 8.05 × 10^−6^ DALYs, followed by atrazine at 1.91 × 10^−6^ DALYs and 2,4-D dimethyl ammonium at 8.93 × 10^−7^ DALYs, respectively. For cancer effects, atrazine also shows a greater effect than acephate at 7.00 × 10^−6^ DALYs and 1.23 × 10^−8^ DALYs, respectively. In upland rice, acephate shows the most obvious health effects (non-cancer) at 1.61 × 10^−6^ DALYs. In terraced rice farming, the health impact was not clearly demonstrated compared to the other areas.

**Figure 4:**
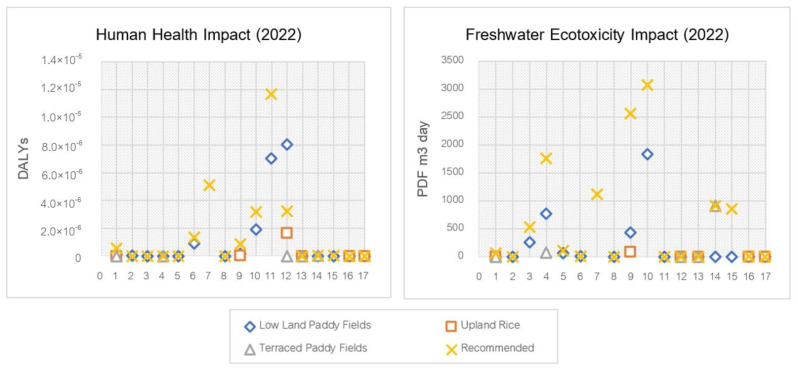
Human health and ecotoxicity impact scores on pesticide use in rice cultivation in 2022. 1. Chlorpyrifos, 2. Glyphosate, 3. Acephate, 4. Alachlor, 5. Butachlor, 6. 2,4-D dimethyl ammonium, 7. Fipronil, 8. Metaldehyde, 9. Mancozeb, 10. Atrazine, 11. Atrazine (cancer), 12. Acephate, 13. Acephate (cancer), 14. Carbendazim, 15. Cypermethrin, 16. Captan, and 17. Captan (cancer).

For freshwater ecotoxicity, lowland rice fields still had the greatest impact due to the intensive use of pesticides by farmers compared to the other areas. Atrazine revealed the greatest impact at 1845.60 PDF.m^3^.day, followed by alachlor at 764.58 PDF.m^3^.day and mancozeb at 429.16 PDF.m^3^.day, respectively. The use of carbendazim in terraced rice farming resulted in a more pronounced effect than other substances at 913.31 PDF.m^3^.day. However, the upland rice area has less freshwater ecotoxicity impact compared to other areas. The health and freshwater ecotoxicity impacts in 2022 are shown in Figure 4.

### 3.7. Pesticide Emission Distribution

**Figure 5:**
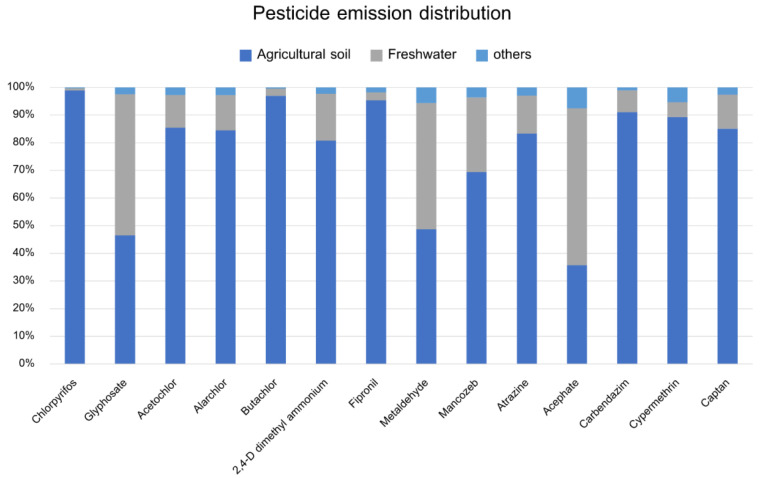
Pesticide emission distribution.

The emission distribution of each pesticide after being released from the agricultural soil is shown in Figure 5. The USEtox model demonstrates that the distribution of pesticides to various environmental media, especially the distribution into freshwater, has been linked to ecotoxicity, coupled with the chemical properties of the compounds. The results showed that most of the pesticides remaining in agricultural soils were chlorpyrifos (98.88%), butachlor (96.94%), and fipronil (95.33%), respectively. The substances with the greatest distributions in freshwater were acephate (56.74%), glyphosate (50.90%), and metaldehyde (45.65%), respectively.

## 4. Discussion

### 4.1. Environmental Impact under Different Rice Cultivation Systems

The more agricultural inputs were used, the higher GHG emissions were generated, which was the case in the present study. Lowland rice cultivation used the largest agricultural inputs compared to the other two rice systems, especially using gasoline and chemical fertilizers, whereas the lowest usage of inputs was found in upland rice cultivation (Table 1). This is because lowland rice is generally practiced as intensive cultivation in flat areas, aiming for maximum economic benefit, whereas upland rice was grown for household consumption. As also presented in Table 3, chemical fertilizers contributed to the highest GHG emission compared to the other inputs at the raw materials production stage, obviously in lowland rice cultivation. Similarly, herbicides and pesticides generated the second and third places in contributing GHG emissions in lowland rice and terraced rice at the raw materials production stage, which is consistent with the studies of Khoshnevisan et al. [9] and Arunrat et al. [42].

Under the anaerobic conditions of flooded paddy fields, the organic matter decomposition by methanogenic bacteria causes CH_4_ emission [43]. It is found in lowland and terraced rice cultivation systems in the present study, where CH4 emission was generated at the highest level compared to the other activities, whereas zero CH4 emission was produced from upland rice fields due to cultivation in dryland areas (Table 3). Alam et al. [44] also reported that roughly 60% of the total GHG emissions from rice production were CH_4_ due to continuously flooded conditions. However, this condition can prevent weed growth and water stress in paddy fields [15], as well as increase sequestered SOC [45]. N_2_O emissions are largely emitted from using chemical fertilizers [46], which is in line with the findings of the present study (Table 3). In the net GHG emission, lowland rice cultivation generated the highest GHG emissions, followed by the terraced and upland rice cultivation systems, which is similar to the rice yield (Table 3). This is because practicing intensive cultivation as lowland rice farming can promote a high yield by using chemical fertilizers to enhance plant growth and applying pesticides and herbicides to prevent weed and pest damage.

Terraced rice cultivation has the highest carbon footprint intensity (1.15 kg CO_2_eq kg^−1^ rice yield), while the lowest was detected in upland rice (0.17 kg CO_2_eq kg^−1^ rice yield) (Table 3). A study by Champrasert et al. [47] estimated the carbon footprint intensity of upland rice in Karen and Lawa tribes in Northern Thailand, with the values of 0.13 and 0.19 kg CO_2_eq kg^−1^ nonmilled rice, respectively. As suggested by several studies [10,13,14,15,48,49], accounting for SOC sequestration in the carbon footprint calculation can increase the accuracy of the net carbon footprint, because farming practices directly affect soil carbon changes. This is also evidenced in the present study that SOC sequestration (ΔSOCS) increased during the study period (Table 2). Adamu et al. [50] mentioned that even a small increase in SOC stock can play an important role in GHG reduction in the atmosphere. Similar to the study by Pan et al. [11], paddy fields have a higher potential to store carbon in the soil than croplands due to the inputs from rice residue, turnover of roots, and root exudates [51]. Therefore, the present study also suggests including the change of SOC sequestration for estimating the carbon footprint of crop production.

The WF is an effective indicator for measuring the direct and indirect water use in the life cycle of a product. Lowland rice generated the significantly highest total WF compared to terraced and upland rice systems, especially blue and gray WFs (*p* < 0.05) (Table 4) because of the higher use of irrigation water, chemical fertilizers, herbicides, and pesticides. This is consistent with the studies of Galloway and Cowling [52] and Benbi [53]. Increasing the yield and reducing fertilizer, herbicide, and pesticide applications can significantly reduce the total WF, which is supported by the studies of Huang et al. [54] and Zhuo et al. [55].

### 4.2. Human and Ecotoxicological Impacts under Different Rice Cultivation Systems

This study found that trends in health and environmental impacts were unclear over the three-year study. Additionally, the types of pesticides and amounts used vary from year to year. Regarding health impacts, acephate and atrazine demonstrated more pronounced cancer and non-cancerous health impacts than the other pesticides. The non-cancerous health impacts are expressions of the health effects demonstrated by an in vitro toxicology test; considering the effect of the intake dose on the target organs that have not been shown, they potentially cause tumors or cancer cells. The quantitative relationship between doses and chemicals that enter the body through various channels (ingestion and inhalation) can cause incidences of adverse health effects. For freshwater ecotoxicity, atrazine and alachlor are pesticides that should be monitored, as they have shown relatively high impacts (e.g., affects the lipid peroxidation and antioxidant enzyme system on *Channa punctatus* and inhibits the synthesis of long-chain fatty acids [56,57]), the same applies to carbendazim, because two-thirds of the rice planting area started using it in 2021–2022, showing a relatively high impact on freshwater ecotoxicity. Fipronil and cypermethrin are not used in rice cultivation every year, but studies show that their impact on health and freshwater ecotoxicity is quite high compared to other substances in this study, and they are other substances that should be considered if needed in the future. Fipronil can cause neurodegenerative disorders [58] and also affect aquatic ecosystems by affecting the behavior, regeneration, and reproduction of *Girardia tigrina* in water when exposed to chronic exposure [59]. In addition, cypermethrin ecotoxicity demonstrates potential hazards on several fish species (e.g., neurotoxicity, endocrine disruption, effects on proximate body composition, and oxidative stress injury) [60]. The freshwater ecotoxicity impact refers to the fraction of a chemical dissolved in freshwater causing the expression of the effect (EC50: effect concentration, a statistical consideration to estimate the effect 50% of a population (e.g., fish) is affected (e.g., died) in the laboratory, HC50: hazardous concentration of a chemical which 50% of species exposed to a concentration above their EC50, PNEC: predicted no effect concentration, NOEC: no observed effect concentration, etc.). Toxicological values in experimental animals will use the geometric mean of all test species depending on the model database [61]. Although most farmers in the three rice cultivation systems used pesticides according to the instructions on the packaging, the predictive modeling of impacts may further determine the trade-offs between pesticide efficacy and toxicity for both health and the environment.

The proportion of distribution into different media depends on the chemical properties of the substance. These data are obtained from the model’s calculations in steady conditions. The state depends on the chemical properties of each substance, such as molecular mass, partitioning coefficient, solubility (Kow), the partitioning coefficient between organic carbon and water (Koc), and Henry’s law constant, among others. Some substances, such as chlorpyrifos, have low Henry’s law constants, resulting in a proportional variation of their aqueous releases [62]. However, the efficacy of the measure is unclear, because other factors are difficult to determine. The efficacy and spread of pesticides depend on many factors, such as the amount of application in each area, the time of use, and the environmental conditions, including different soil conditions. The adhesion to soil particles is completely different in each soil type with different properties. The pH value is especially related to the degradation of pesticides, which affects the persistence of pesticides in the soil [63]. In addition, the persistence of pesticides depends on the combination of organic matter content and cation exchange capacity, among other factors [64].

However, the limitation of the USEtox model should be mentioned for future development. The life cycle assessment (LCA) and risk assessment are methodologically constrained methods, as they cannot be replicated in some complex environments, such as the breakdown of pesticide active ingredients (metabolism). The effects of a single substance at a steady state can be assessed in the USEtox model. The interaction effects between many chemicals and their metabolisms cannot be considered. The USEtox model considers two main exposure pathways: ingestion and inhalation exposure pathways. Exposure due to the proximity to sources such as farmers spraying pesticides and dermal channels, which are attractive exposure pathways, were not considered. In addition, the USEtox model evaluated the human carcinogenic and noncarcinogenic effects separately, which could not determine the specific illnesses directly related to the pesticide (e.g., lung cancer). The effectiveness of pesticides is influenced by other factors, such as time and level of pesticide application and environmental conditions, such as the characteristics of different soil types in each area, as well as chemical factors (e.g., soil pH and organic matter, among others). We acknowledge that the model depends on the uncertainty of the data, so laboratory results or field experiments are essential to test the robustness of each pesticide model in different cultivation areas. Some pesticides have no toxicological data, and, therefore, could not be calculated in this study, which was another limitation. This study examined the emission pathways of pesticides to agricultural soils, and future studies may use other models that could link the assumptions together to account for other environmental aspects.

## 5. Conclusions

The more agricultural inputs, particularly chemical fertilizers, herbicides, and pesticides, that were used, the more carbon footprint and gray WF were generated. Lowland rice production had the highest net GHG emissions due to intensive practices, followed by terraced and upland rice cultivation systems. In contrast, terraced and upland rice cultivation were grown relying on natural conditions and produced for household consumption. Similarly, the highest WF was found in lowland rice fields compared to terraced and upland rice systems due to the higher use of irrigation water, chemical fertilizers, herbicides, and pesticides. Alachlor, atrazine, and 2,4-D dimethyl ammonium were the top three pesticides used during the studied periods. Acephate and atrazine demonstrated more pronounced cancerous and non-cancerous health impacts than other pesticides. Meanwhile, atrazine, alachlor, and carbendazim should be monitored as relatively high freshwater impacts. The lowland rice fields had the most impacts on human health and freshwater ecotoxicity, followed by the terraced and upland rice cultivation systems. This study indicated that relying on nature-based resources for cultivation as upland rice consumed less anthropogenic sources, resulting in low environmental, human, and ecotoxicological impacts. Overall, the use of pesticides in the studied areas did not exceed the recommendations on the packaging, resulting in low health effects.

## Figures and Tables

**Table 1:** Input inventory of lowland, terraced, and upland rice systems during 2020–2022 (mean ± standard deviation).

Input	Unit	Quantity		
		Lowland	Terraced	Upland
Seeds	kg ha^−1^ crop^−1^	137.5 ± 50.0 ^a^	112.5 ± 31.25 ^a^	125.00 ± 31.25 ^a^
Gasoline	L ha^−1^ crop^−1^	125.0 ± 43.75 ^a^	62.5 ± 31.25 ^b^	0
Diesel	L ha^−1^ crop^−1^	156.25 ± 8.3 ^a^	125.0 ± 31.25 ^a^	0
Straw	kg ha^−1^ crop^−1^	3474.4 ± 1250 ^a^	2618.5 ± 1250 ^b^	1316.7 ± 625 ^c^
Manure	kg ha^−1^ crop^−1^	1250 ± 312.5 ^a^	937.5 ± 187.5 ^a^	0
Labor	man day^−1^ crop^−1^	4.0 ± 2.0 ^a^	5.0 ± 3.0 ^a^	3.0 ± 2.0 ^a^
Fertilizer 16-20-0	kg ha^−1^ crop^−1^	156.3 ± 85.2 ^a^	156.3 ± 75.3 ^a^	0
Fertilizer 16-16-8	kg ha^−1^ crop^−1^	125.0 ± 31.25 ^a^	125.0 ± 31.25 ^a^	62.5 ± 37.25 ^b^
Fertilizer 46-0-0	kg ha^−1^ crop^−1^	62.5 ± 37.25	0	0

^a–c^ represent significant differences in inputs among rice systems (*p* < 0.05).

**Table 2:** SOC stock at 0–30 cm, and the SOC sequestration rate from 2020–2022 (mean ± standard deviation).

Rice System	SOC 2020 (kg C ha^−1^)	SOC 2021 (kg C ha^−1^)	SOC 2022 (kg C ha^−1^)	ΔSOCS (kg C ha^−1^ year^−1^)	ΔSOCS (kg CO_2_ ha^−1^ year^−1^)
Upland	61,032.0 ± 1107 ^aA^	61,093.6 ± 1171 ^aA^	61,068.6 ± 867 ^aA^	36.6 ± 8.6 ^A^	134.3 ± 32.1 ^A^
Terraced	44,933.7 ± 1312 ^aB^	44,991.3 ± 1208 ^aB^	44,960.5 ± 1045 ^aB^	26.7 ± 10.1 ^A^	98.1 ± 38.9 ^A^
Lowland	32,751.7 ± 978 ^aC^	32,782.6 ± 1087 ^aC^	32,804.7 ± 1127 ^aC^	52.9 ± 12.2 ^B^	194.0 ± 45.1 ^B^

^a^ represents significant differences in SOC stocks among years 2020, 2021, and 2022 (*p* < 0.05). ^A–C^ represents significant differences in SOC stocks among rice systems (*p* < 0.05).

**Table 3:** GHG emissions of lowland, terraced, and upland rice systems during 2020–2022 (mean ± standard deviation).

Life Cycle Stage		Rice System		
		Lowland	Terraced	Upland
Raw material production (kg CO_2_eq ha^−1^ year^−1^)	Seeds	24.5 ± 0.5 ^a^	22.3 ± 0.5 ^a^	31.3 ± 2.5 ^a^
	Gasoline	58.7 ± 1.2 ^a^	22.1 ± 0.6 ^b^	0
	Diesel	38.5 ± 1.5 ^a^	17.8 ± 1.1 ^b^	0
	Chemical fertilizers	686.5 ± 181.5 ^a^	315.6 ± 93.6 ^b^	125.7 ± 24.6 ^c^
	Herbicides	82.4 ± 10.3 ^a^	58.3 ± 8.5 ^b^	35.5 ± 5.5 ^c^
	Pesticides	58.6 ± 7.6 ^a^	97.5 ± 10.1 ^b^	42.4 ± 8.4 ^a^
Field emission (kg CO_2_eq ha^−1^ year^−1^)	Gasoline	312.4 ± 5.7 ^a^	167.5 ± 3.8 ^b^	0
	Diesel	257.7 ± 7.7 ^a^	113.1 ± 4.1 ^b^	0
	Herbicides	98.7 ± 0.7 ^a^	65.3 ± 1.1 ^b^	42.3 ± 0.4 ^c^
	Pesticides	26.6 ± 1.4 ^a^	83.5 ± 1.7 ^b^	21.1 ± 0.6 ^a^
	CH_4_	2767.9 ± 1512.4 ^a^	2914.3 ± 1604.5 ^a^	0
	N_2_O	316.8 ± 21.3 ^a^	189.7 ± 15.4 ^b^	97.5 ± 11.3 ^c^
	Labor	77.4 ± 5.4 ^a^	51.6 ± 3.8 ^b^	38.7 ± 10.4 ^c^
	Harvesting	121.3 ± 15.2	0	0
Total GHG (kg CO_2_eq ha^−1^ year^−1^)		4928.0 ± 2132.5 ^a^	4118.6 ± 1976.4 ^b^	434.5 ± 101.5 ^c^
Net GHG emissions (kg CO_2_eq ha^−1^ year^−1^)		4734.0 ± 1042.5 ^a^	4020.5 ± 867.5 ^b^	300.2 ± 87.6 ^c^
Yield (kg ha^−1^ year^−1^)		4632.5 ± 870.6 ^a^	3491.3 ± 367.5 ^b^	1755.6 ± 750.5 ^c^
Carbon footprint intensity (kg CO_2_eq kg^−1^ rice yield)		1.02 ± 0.32 ^a^	1.15 ± 0.66 ^b^	0.17 ± 0.09 ^c^

^a–c^ represent significant differences in GHG emissions among rice systems (*p* < 0.05).

**Table 4:** Water footprint of the lowland, terraced, and upland rice systems during 2020–2022 (mean ± standard deviation).

Rice System	Green WF (m^3^ ton^−1^)	(%)	Blue WF (m^3^ ton^−1^)	(%)	Gray WF (m^3^ ton^−1^)	(%)	Total (m^3^ ton^−1^)
Upland	1217.9 ± 108.9 ^a^	94.9	0.0	0.0	65.3 ± 17.6 ^a^	5.1	1283.2 ± 121.3 ^a^
Terraced	1116.8 ± 112.3 ^a^	78.5	37.5 ± 12.5 ^a^	2.6	267.8 ± 100.3 ^b^	18.8	1422.1 ± 135.8 ^a^
Lowland	1008.5 ± 110.1 ^a^	59.3	103.4 ± 56.8 ^b^	6.1	589.7 ± 125.7 ^c^	34.7	1701.6 ± 181.4 ^b^

^a–c^ represent significant differences in the water footprint among rice systems (*p* < 0.05).

**Table 5:** Type and amount of pesticide used by farmers in each area.

Rice Cultivation System	Pesticides	Average a.i. Use ± S.D. (kg ha^−1^ year^−1^) 2020	Average a.i. Use ± S.D. (kg ha^−1^ year^−1^) 2021	Average a.i. Use ± S.D. (kg ha^−1^ year^−1^) 2022	Average Active Ingredients Recommended (kg ha^−1^ year^−1^)
Lowland	2,4-D	0.919 ± 0.275	1.160 ± 0.236	1.116 ± 0.336	1.680
	Acephate	0.547 ± 0.135	0.498 ± 0.150	0.469 ± 0.149	0.188
	Acetochlor	0.605 ± 0.270	0.692 ± 0.235	0.750 ± 0.264	1.563
	Alachlor	1.400 ± 0.346	1.586 ± 0.285	1.560 ± 0.329	3.600
	Atrazine	1.246 ± 0.724	1.256 ± 0.530	1.350 ± 0.605	2.530
	Butachlor	0.984 ± 0.600	1.055 ± 0.374	1.125 ± 0.410	1.800
	Chlorpyrifos	0.125 ± 0	0.143 ± 0.0001	0.125 ± 0	1.000
	Fipronil	-	0.125 ± 0	-	0.050
	Glyphosate	0.881 ± 0.442	0.793 ± 0.266	0.990 ± 0.376	0.450
	Mancozeb	0.500 ± 0	0.500 ± 0	0.500 ± 0	3.000
	Metaldehyde	0.536 ± 0.152	0.564 ± 0.143	0.577 ± 0.117	1.313
Upland	Acephate	-	-	0.0002 ± 0	0.188
	Alachlor	0.15 ± 0	0.09 ± 0	-	3.600
	Carbendazim	0.312 ± 0	0.188 ± 0	-	0.188
	Chlorpyrifos	-	-	0.025 ± 0	1.000
	Cypermethrin	-	0.131 ± 0	-	0.044
	Mancozeb	-	-	0.0002 ± 0	3.000
Terraced	2,4-D	0.525 ± 0	0.525 ± 0	-	1.680
	Alachlor	-	-	0.150 ± 0	3.600
	Carbendazim	0.250 ± 0.088	0.312 ± 0	0.188 ± 0.177	0.188
	Captan	-	0.938 ± 0	-	0.125
	Chlorpyrifos	-	-	0.013 ± 0	1.000
	Cypermethrin	0.109 ± 0	-	-	0.043
	Fipronil	-	0.125 ± 0	-	0.050
	Mancozeb	0.500 ± 0	0.500 ± 0	-	3.000
	Metaldehyde	0.313 ± 0	0.313 ± 0	-	0.313

## Data Availability

Not applicable.
